# Information and support to patients when the waiting time guarantee cannot be fulfilled: a qualitative study

**DOI:** 10.1186/s12939-023-01935-1

**Published:** 2023-07-10

**Authors:** Belén Casales Morici, Hanna Augustsson, Henna Hasson, David Ebbevi

**Affiliations:** 1 Department of Learning, Informatics, Management and Ethics, Medical Management Centre, Karolinska Institutet, Stockholm, Sweden; 2 Department of Business Studies, Uppsala University, Uppsala, Sweden; 3 Unit for Implementation and Evaluation, Center for Epidemiology and Community Medicine (CES), Region Stockholm, Sweden; 4 Department of Global Public Health, Karolinska Institutet, Stockholm, Sweden; 5 Astrid Lindgren Children’s Hospital, Karolinska University Hospital, Stockholm, Sweden

**Keywords:** Health literacy, Health services accessibility, Reimbursement mechanisms, Waiting times

## Abstract

**Background:**

Long waiting times for health care services are a prominent health policy issue. Waiting time guarantees may limit time to assessment and treatment.

**Methods:**

This study aims to investigate the information and support given to patients when the waiting time guarantee cannot be fulfilled from a care provider and administrative management perspective. Semi-structured interviews (*N* = 28) were conducted with administrative management and care providers (clinic staff and clinic line managers) in specialized clinics in the Stockholm Region, Sweden. Clinics were purposefully sampled for maximum variation in ownership (private, public), complexity of care, geographical location, volume of production, and waiting times. Thematic analysis was applied.

**Results:**

Care providers reported that patients received inconsistent information and support with regard to the waiting time guarantee and that information was not adapted to health literacy or individual patient needs. Contrary to local law, they made some patients responsible for finding a new care provider or arranging a new referral. Furthermore, financial interests affected whether patients were referred to other providers. Administrative management steered care providers’ informing practices at specific time points (upon establishment of a new unit and after six months of operation). A specific regional support function, Region Stockholm’s Care Guarantee Office, helped patients change care providers when long waiting times occurred. However, administrative management perceived that there was no established routine to assist care providers in informing patients.

**Conclusions:**

Care providers did not consider patients’ health literacy when informing them about the waiting time guarantee. Administrative management’s attempts to provide information and support to care providers are not producing the results they expect. Soft-law regulations and care contracts seem insufficient, and economic mechanisms undermine care providers’ willingness to inform patients. The described actions are unable to mitigate the inequality in health care that arises from differences in care-seeking behavior.

## Background

Long waiting times for healthcare services are a prominent health policy issue [1] that impact patients’ health, quality of care, and trust in care. Waiting-time guarantees are a common solution for tackling excessive waiting times. These policies state patients’ right to receive care within a certain time and are often formulated as a pre-determined maximum waiting time for patients. The objective of waiting time guarantees is to provide care on equal terms and within an appropriate time to the population and to ensure that healthcare is accessible for those in need [1].

Maximum waiting time guarantees are increasingly used in countries with tax-based systems, including Sweden, Finland, Denmark, Norway, England, Scotland, Italy, Spain, Canada, New Zealand, and Portugal. The waiting time guarantee policies differ widely across countries in the length of time limits and in how the policy is formulated [1]. Some countries apply strong sanctions or economic incentives or enable patients to use an alternative provider when waiting time limits are exceeded [2]. Several countries measure waiting times, but there is variation in what is measured (ongoing or completed waits), the parameters employed (median or mean number of days), with which activity measurement begins, and the types of patients and healthcare units that the data concern.

Since it may be difficult for patients to exercise their rights stated in the waiting time guarantees, a critical issue is how the guarantees are enforced. Commonly, this is done through active administrative processes or by allowing patients to choose alternate providers [1]. Patients’ knowledge of the waiting time guarantee and their right to change care providers can be assumed to support patients’ freedom of choice. Therefore, it is essential that patients receive individually adapted information about their rights and how to navigate the health system. Due to the information asymmetry between care providers and patients [3], patients depend on care providers’ skills, resources, and decisions to be able to participate in their care and exercise their freedom of choice [3, 4]. This has been widely acknowledged regarding diagnosis, treatment, and rehabilitation [4], but also applies to waiting times [5].

Several studies indicate that patients are not well informed by care providers about the waiting time guarantee [6, 7]. Known causes are care providers’ disincentives to offer information and the inherent methodological difficulties of providing valid and updated information on waiting times in a relevant and understandable format for the patient [8, 9]. Previous research has investigated the extent to which waiting time requirements are followed [10], current waiting time policy designs and their effect on patient participation [8], the relationship between waiting times and hospital service satisfaction [7], and healthcare providers and administrative management professionals’ perceptions of the validity and usefulness of waiting times [11]. However, they have not elucidated information and support provided to patients concerning waiting-time guarantees and the system conditions influencing this.

## Methods

This study aims to investigate the information and support given to patients when the waiting time guarantee cannot be fulfilled from a care provider and administrative management perspective.

### Design

A qualitative interview study was conducted to explore how health care administrators and providers inform and support patients when the waiting time guarantee is not fulfilled. We have followed the Consolidated Criteria for Reporting Qualitative Studies (COREQ) [12] and Standards for Reporting Qualitative Research (SRQR) [13] when reporting the study. Checklists are provided in Appendix 1.

### Setting

The study was conducted in specialist care in Region Stockholm, Sweden’s largest health care provider organization. Swedish health care is largely publicly funded, where public or private care providers share the same financing model, and the patient fees are low. In Region Stockholm, the care is organized in a provider/purchaser model, i.e., care provision is separate from care administration. In this study, the two groups studied are hereafter called care providers and administrative management. Specialist care is defined as specialized healthcare that cannot be provided in primary care, for instance, ophthalmology, psychiatry, urology, pediatrics, etc. Stockholm has a high degree of specialist care providers organized under a care choice system. This system has two components, the right of providers to freely establish new care providers as they see fit, and the right of the patient to freely choose a care provider. For some specialties this is combined with the freedom to seek care without referral from primary care (i.e. self-referral). 29% of health care in Region Stockholm was privately owned at the time of data collection (the highest degree in all regions of Sweden). Further, Stockholm has a high consumption of specialized care with 1,6 visits per inhabitant the year of data collection (32% more than the national average).

In Sweden, maximum waiting times for first appointment and treatments are stipulated by national legislation [14]. Local policies can require even shorter waiting times for regional care providers. During this study, Region Stockholm’s waiting time guarantee allowed a maximum of 30 days to get a specialist care visit and 90 days for a specialist procedure. This applies both to referrals from care providers and patient self-referral to specialized care. Exceptions can be made if the patient chooses to change care provider for the same health problem or decides to waive the waiting time guarantee (patient-selected waiting). Activities in specialized care that cannot maintain the time limits of the waiting time guarantee are reported to the specialist clinic’s contract administrator through a computerized system.

The care provider is obliged to notify the patient about long waiting times and refer the patient to a care provider with a shorter waiting time or to Region Stockholm’s Care Guarantee Office. This should be done as soon as they realize that they are not able to offer care within the care guarantee. The obligation to inform patients about the waiting time guarantee is statutory in accordance with the Patient Act [14] (2014: 821) and the Health and Medical Care Act [15] (2017:30). They state that care providers must offer information and support to patients about the waiting-time guarantee and the options available. Furthermore, patients have the right to receive personalized information, i.e., the information must be adapted to the recipient’s age, maturity, experience, linguistic background, and other individual conditions [14].

### Participants

The informants included care providers and administrative management. To sample care providers, we used a maximum variation strategy [16] and included clinics based on the complexity of care, ownership (i.e., private or public), the capacity of production, geographical location, and attainment of the waiting time guarantee (data from September 1, 2017). Final sample had six secondary health care units, two tertiary and one quaternary; five were public and four private, four were located north of Stockholm, four south and one in central Stockholm; attainment to the care guarantee ranged from 40 to 100%, mean was 75% and standard deviation 24%.

Care provider informants included two types of staff: 1) clinic staff responsible for reporting to the waiting time registry and the supply service database (*n* = 8) and clinic line managers (*n* = 9). Twenty nine clinics were contacted via managers, and 20 declined to participate. Twenty one were first invited, and based on the characteristic of those that declined an additional eight were invited to align with the maximum variation strategy. In most cases, they did not respond (*n* = 14). Others said they had nothing to say about the waiting time guarantee (*n* = 1), their care processes were not included the waiting time guarantee (*n* = 1), they did not have time to conduct the interview (*n* = 1), or left no explanation for declining participation (*n* = 3). Eight clinics participated represented by a clinic manager and a clinic staff member. One clinic participated with a clinic manager but no clinic staff member. Clinic staff were recruited by their line managers at the participating care unit. We invited informants through email, and those who did not reply were contacted by telephone.

Regarding participants from the administrative management, their main tasks include (1) a contractual and auditing function where they assess compliance to the waiting time guarantee and (2) the quality assessment of the administration and development of the waiting times registry. As a starting point, five administrative managers were contacted upon the suggestion of the administrative management that provisioned the study. In addition, nine managers were identified by snowball sampling. Three informants declined to participate, stating that they had nothing to say about the issue. In total, 17 care provider informants and 11 administrative management informants were included in the study.

### Data collection

The interviews were conducted between October 2017 and January 2018. A semi-structured interview guide was used [16]. To construct the first draft of the interview guide, discussions with personnel working with administrative management (two of whom were later interviewed) and similar studies were considered [6, 9, 17–19]. The interview guide was revised iteratively and covered information provided about patient rights within the framework of the waiting time guarantee. The questions were tailored to the functions of the two groups of informants. For instance, questions inquired how care providers inform patients about their rights within the framework of the waiting time guarantee, through which channels, what information is provided, perceived connections between waiting times and information given, examples of when clarification concerning waiting times was needed, common misunderstandings, strategies for countering misunderstandings, and use of written material. Additional questions outside the scope of the current study concerned perceptions of the validity and usefulness of waiting times, and this has been published elsewhere [11]. Sampling of informants in the two groups continued until data saturation was achieved. All informants gave written informed consent. At the request of the informants, three were interviewed in one group, and six were interviewed in groups of two (only participants in the same unit). The interviews were conducted by the second and the last authors, who have extensive experience in conducting research interviews. The interviews ranged from 23 to 71 min and were conducted at the informants’ workplaces to promote a familiar environment. Audio was recorded and field notes were taken.

### Data analysis

The interviews were transcribed verbatim by an external transcriber and analyzed to capture manifest content using thematic analysis [20]. See Appendix 2 for a further description of the analytic steps. The interview transcripts were analyzed using NVivo (v.12 Pro). The analysis started with the first author reading and re-reading the data several times and, in the next step, creating inductive codes.

When a stable coding system was obtained, the codes were reviewed by the first and second authors. Both authors worked through the codes, scanning everything relevant to the research question. When faced with ambiguities and discrepancies, differences in the coding between the authors were discussed. As both authors worked through this joint analysis, new codes were generated and re-defined. After the codes had been created, these were grouped into preliminary themes and subthemes. Themes were created iteratively, moving back and forth between the analysis steps. We re-read the themes and subthemes several times to assess whether they were supported by the data and to make sure the themes were coherent and distinct from each other. Two segments of text co-occur in two themes.

## Results

The results will be presented in two parts. The first part concerns the care providers’ information and support to patients when the waiting time guarantee could not be fulfilled and factors influencing this. The second part includes the action taken by the administrative management to make sure that care providers offer the appropriate information to patients.

### Care providers’ information and support to patients when the waiting time guarantee was not fulfilled

The results for the first part entailed three different themes highlighting: (1) Inconsistent support to patients, (2) Inconsistent information to patients, and (3) Financial disincentives for efficient referral of patients (Table 1).

**Table 1:** Care providers’ information and support to patients when the waiting time guarantee was not fulfilled

Theme	Subtheme
Inconsistent support to patients	▪ The patient is responsible for a new referral
	▪ The care provider is responsible for a new referral
Inconsistent information to patients	▪ Patients are assumed to find information themselves
	▪ Information is provided to all patients
	▪ Information is provided to specific patients
	▪ Patients do not receive information
Financial disincentives for efficient referral of patients	

#### Inconsistent support to patients

Care providers reported inconsistent support to patients when the waiting time guarantee was not fulfilled. There was variation both across providers and across patients for the same providers. The difference across patients was not explained by individual patients need for support but rather that the waiting times became longer than they usually were. In some cases, the patient had to seek information and a new referral, while in other cases, the specialist care provider took responsibility for referring the patient to another provider.

#### The patient is responsible for a new referral

The responsibility of finding another care provider when the current provider could not provide care within the waiting time guarantee limit was sometimes allocated to the patients. The head of the clinic described the situation as follows:

When the patient asks: ‘Can I turn elsewhere? Can you refer me elsewhere?’ We say ‘No, we cannot refer you elsewhere; we do not have an agreement for neuropsychiatric investigations. However, you can turn to other clinics yourself.’

This was done in three main ways. One way for patients to find a new care provider was to contact the Care Guarantee Office, which helps patients find a provider with a shorter waiting time. Another way was to contact the specialist that did not fulfill the waiting time guarantee, asking them to remove the referral and send a new referral to another specialist. Finally, a third way was to contact the referring physician and ask them to send a new referral. Care providers acknowledged that putting the responsibility on patients to get a new referral made it difficult for patients. A manager at a specialist care center explained this as follows:

They can contact us and say that we should remove the referral from here, or the referrer will do it, or they may well send the referral elsewhere. Or ask us to write it elsewhere. … So it’s a bit complicated for the patient.

#### The care provider is responsible for a new referral

In other cases, the care provider took the responsibility of sending a new referral to another provider when the waiting time guarantee time could not be fulfilled. The patient was informed and could suggest where the new referral should be sent. Established collaboration between care providers facilitated the sending of referrals between care providers, i.e., when a care provider could not offer an appointment within the waiting time guarantee limit, they sent the referral to other providers with which they collaborate. These collaborations existed both between and within corporate groups or administrative organizations. In line with this, a secretary from a care unit described the following:

I have, on some occasions, had very long waiting times, and then we had a collaboration with two different care centers. It was on two different occasions, and then we called those who had waited a long time and told them that ‘We will not be able to fulfill the waiting time guarantee. However, we have a collaboration with this care center; if you want, we can move the referral there. They have promised to accept’ … Then, many patients took up on that offer, and we sent the referrals ... Others said ‘No, we would rather wait.’

#### Inconsistent information to patients

According to the care providers, the information given to patients about the waiting time guarantee was diverse. There was variation both across providers and across patients for some providers. This varied from no information, patients needing to find the information themselves, general information provided by the care provider to all patients, or information provided to specific patients. If patients were actively informed, they were informed at the time of booking.

#### Patients are assumed to find information themselves

Informants described how patients did not receive information from them about the waiting time guarantee and that patients were expected to find information themselves. At the same time, it was not always clear how patients could get this information. A secretary from a care unit said:

Patients get no information directly from us. I think they contact [the healthcare guide] 1177.

One source of information from which care providers suggested that patients could find information about the waiting time guarantee was the service offered by the *healthcare guide 1177*, the regions’ service for care advice. Its advice can be obtained on the web and by telephone and includes information about what happens if the patient does not receive care within the waiting time guarantee and the current waiting times in all regions of Sweden.

#### Information is provided to all patients

Some of the care providers offered standardized information in brochures and pamphlets that were available at the care center reception or on the care center website. These included general information about the waiting time guarantee and where to turn if care could not be provided within the waiting time guarantee limits. A care unit operation manager explained:

We have a standard summons for everyone. Then, if a patient asks for certain things we can send information about that thing, but the information is the same

#### Information is provided to specific patients

In other cases, patients were informed directly by the care provider about their inability to meet the waiting time guarantee and where the referral will be sent. The information was provided by letters in which patients were informed about estimated waiting time, their right to use the waiting time guarantee and where to turn to exercise it. It also included information about the possibility to remain on the waiting list. However, no informant described adjusting information to the individual needs of the patient. A care unit administrator said:

If we cannot offer a time, then we explain to the patient that we have received a referral from his/her doctor and, unfortunately, we cannot offer an appointment within this number of days, we have such long waiting times… ‘You can turn here and here if you want another appointment; otherwise, contact us, and we will put you on the waiting list.’ Then they can answer and get back to us.

#### Patients do not receive information

Some care providers said that patients did not receive any information from them about the waiting time guarantee. Sometimes this was simply because the care provider did not have long waiting times, and thus there was no need to inform. However, lack of time or unawareness about the obligation to inform the patient and what information should be provided were also reasons for not informing patients. A care unit manager described an attempt to inform their patients about where to turn to get help concerning the waiting time guarantee by including contact information to the Care Guarantee Office in all their summonses. This initiative resulted in many calls to the Care Guarantee Office, and the care provider was therefore asked to remove the information from the standardized summonses:

We had the information in our summonses, we had the care guarantee office’s telephone number. Then we were called by them; they thought we should remove it because they got many calls from our patients, so we removed it.

#### Financial disincentives for efficient referral of patients

Care providers described how the financial system hindered the efficient referral of patients within the healthcare system. To ensure correct cost allocation, specialists sometimes had to send patients back to their general practitioner instead of sending the referral directly to another provider. This way they did not receive an invoice for the requested test. The procedure required several unnecessary steps and caused unnecessary visits to primary healthcare. A care unit manager described this situation as an unnecessary loop for everyone involved:

When we have patients in the emergency room, young, healthy, but we think they should do a gastroscopy, they do not need to do it at the hospital, but if we send the referral, we get an invoice. So we must ask patients to contact their general practitioner and say that they have had a stomachache, and we recommend that they do a gastroscopy … But it is unnecessary to take the general practitioners’ resources, who are already overloaded … “Why can we in the emergency room not say, ‘Now I’ll write a referral to that unit’ and they set up the referral without asking us to pay for it? Because if we must pay for it, we rather do it ourselves. I think it is a great pity for the healthcare system that it must move through this loop

Another example was care providers’ unwillingness to refer patients to other care providers outside their corporate system. Internal instructions from company management hindered physicians from referring patients to other care providers despite not being able to meet the waiting time guarantee limit. However, this example was described by an informant from another care provider. The informant described how an individual physician working in such a system who wanted to send patients elsewhere provided the patient with a referral on paper because an electronic referral would be tracked in the IT system:

Surgery has had unacceptably long waiting times at [a competing provider], … maybe five and eight months of waiting … We had meetings at the surgical clinic with [the provider] where we suggested that we could take some of their referred patients, but they totally refused … I think they had, within [the provider], a system where their healthcare centers are forced to refer to their own units. There are many patients who have come to us through detours who have told us about it.

### Administrative management’s actions to ensure that care providers offer the required information to patients

The results for the second part, outlining the action taken by administrative management to ensure that care providers offer appropriate information to patients, entailed three themes: (1) impose requirements on care providers to deliver information, (2) patient guidance and support through the Care Guarantee Office, and (3) lack of systematic guidance to care providers (Table 2).

**Table 2:** Administrative management’s actions to ensure that care providers offer the required information to patients

Themes
Impose requirements on care providers to deliver information
Patient guidance and support through the Care Guarantee Office
Lack of systematic guidance to care providers

#### Impose requirements on care providers to deliver information

Care providers’ responsibility to inform about the waiting time guarantee and the patients’ options for choosing another care provider is established in the contractual agreement for the provision of healthcare. The agreement states that the care provider must assist patients in contacting another care provider when needed. Administrative management described two different ways they used to supervise care providers in fulfilling their information requirements to patients when the waiting time guarantee was not met: informing care providers about their obligation to provide information and monitoring.

The administrative management stated they had an ongoing dialogue with care providers about their responsibility to provide relevant information to patients about the waiting time guarantee. In this dialogue, the administrative management emphasized their obligation to inform patients about their right to choose another specialist if the waiting time guarantee could not be fulfilled. One informant from the administrative management explained the following:

We often talk to the care providers about this, saying that they must be clear when informing about the waiting time guarantee…‘How do you inform patients of their right regarding the waiting time guarantee?’ Because patients have the right to go to another care provider.

Administrative management also arranged informational meetings with newly contracted care providers. During these meetings, they emphasized the importance of providing information to patients about the waiting time guarantee:

We have startup meetings when the care providers are to start where we talk a lot about the importance of informing the patient and that they need to have a structured way to do that. So they do get the information that they have to do it, but it’s only at one point, when we’re just about to start.

The administrative managers infrequently conducted follow-up with care providers concerning their information to patients about the waiting time guarantee. This monitoring was conducted on an as-needed basis when there were indications of a problem, for example, if the care providers had long waiting times and/or they were not informing patients about the waiting time guarantee. From these follow-ups, it had become obvious to the administrative managers that the awareness of the obligation to inform about the waiting time guarantee and the extent to which care providers informed patients varied between care providers. This was believed, at least in part, to be explained by the many different priorities managers have.

And then when I talked to individual managers, it became clear that some of them had informed about the waiting time guarantee and others had not. Because if the patient should be able to benefit from the waiting time guarantee, they need to invoke it, but if you do not even know that it exists, you cannot invoke it. So I discovered that it differed, how they had informed, if they had routines around it or not. When we talked about it, it got better, but then there are new managers, and they have an awful lot of things to keep track of.

Administrative management also conducted predetermined follow-ups on how specialist care providers informed and supported patients concerning waiting time guarantee issues six months after their contract started. This was conducted during a visit to the new care centers with the aim of making sure that everything was working satisfactorily, including provision of information to patients about the waiting time guarantee:

We usually have that question as a standing question. We perform follow-ups of these care units six months after they have started. We visit the care provider and do a check, a so-called six-month follow-up, to make sure that they have got started and that everything goes as it should. Then one question is if they inform patients about the waiting time guarantee and how they handle this. Then everyone answers, ‘Yes, of course,’ but obviously, not everyone does it.

Although these meetings represented a structured follow-up that was intended to be performed for all new care providers, these follow-ups had not been conducted for quite some time, according to an informant.

#### Patient’s guidance and support through the Care Guarantee Office

Administrative management also provided support directly to patients through the Care Guarantee Office. The responsibility of this office was to help patients who were not offered care within the limits of the waiting time guarantee to find a care provider with shorter waiting times. For the Care Guarantee Office to provide this support, care providers were required to report waiting time forecasts with preliminary estimations to administrative management. This data was then compiled in online reports and used by the Care Guarantee Office as a basis for finding care providers with the shortest waiting times. The reports could also be used by care providers or contract managers who wished to get a better overview of waiting times. An informant from administrative management stated:

I compile the waiting times of specialist care providers, outpatient visits treatment, etc., which I send out. I take out information about waiting times if it is needed either for a single care provider or if there is a group that wants to look at their waiting times or if a contract manager wants to look at their caregivers. I send monthly reports to management.

#### Lack of systematic guidance to care providers

Despite the actions described above, administrative managers stated that there was no established routine or procedure to support and monitor care providers’ information and support to patients concerning the waiting time guarantee. An informant from administrative management stated:

I do not think that there is guidance or routine on how to inform patients…You should be aware of the existence of the waiting time guarantee.

Administrative managers described being aware of care providers informing their patients about the waiting time guarantee in different ways and to different extents. At the same time, they also expressed being unaware of how care providers fulfilled the information requirements, indicating a lack of systematic follow-up for the issue.

When we ask care providers how they inform about the waiting time guarantee, some of them say ‘yes, we inform about that’. Yes, but when? ‘Yes, we say it when patients call’… However, there is not any system behind it. While others say: ‘the waiting time guarantee is very clear in our summons, patients can choose this or that or contact the Care Guarantee Office’.

I only know that care providers should inform about the waiting time guarantee when they book patients and [rebook]… If the patient wants to see a certain doctor, they should be informed that the waiting time guarantee does not apply and so on, but if they live up to it, I have no idea

Informants from administrative management believed that a possible consequence of the limited and inconsistent information and support to patients was that patients were unaware of their rights to a waiting time guarantee:

The population does not know what care choice system is. We have failed to convey that message, which actually means that… You may not know that you can choose another care provider. I actually think that is quite common. It is very important that the care providers inform that you can choose from these 40 [providers] as well, go ahead.

## Discussion

The findings of this study show inconsistency in the information and support provided to patients and the responsibility put on patients in relation to the waiting time guarantee. This variation is not explained by a perceived difference in patients’ ability or need for information, i.e., patients in great need of information or support are not receiving it properly. Instead, other mechanisms seem to control the amount of information and support given. For example, a lack of guidance or monitoring from the administrative management and financial structures hinder need-adjusted information and support.

Patient tailored instructions are important because individuals’ ability to process symptoms and care instructions and to navigate health care systems (i.e., health literacy) differ [3]. The uneven distribution of health literacy can be countered through customized instructions and interactions such as person-centered approaches or tailored communication [21]. Population equality in health depends on the healthcare system’s ability to meet this demand [22]. However, the findings of the present study contain no account of information adjusted to the needs of a patient and several accounts of care providers that give no information about the waiting time guarantee. Of course, a qualitative maximum variation sampling study cannot assert that something does *not* occur. However, it does lend support to that notion, and it is likely that the current system is unable to mitigate the inequality in health that arises from differences in care-seeking behavior.

The finding that many patients receive no information about the waiting time guarantee aligns with findings from previous studies in 2010 [23] and 2014 [24]. The finding that the degree of information and provided support varies between care providers agrees with an earlier study on the waiting time guarantee in Sweden [25]. That study used a purposive sample from other regions than Stockholm and was performed in 2015. It seems that little has changed since these studies were performed, and it is reasonable to look for explanations at the administrative management level for the lack of tailored information and support to patients. Recognition of the reasons for not providing information is important in identifying effective strategies to overcome them.

Previous studies that focus on reasons for not providing information about waiting times have limited explicability. Studies focusing on patient harm have found that information was not given to patients due to workload and time pressures and a lack of correct written material, embedded information, and communication technology systems [26]. The studies have not clarified how administrative functions or structural conditions contribute to information and support practices—with a few exceptions where staffing was studied [27]. Here, our study finds mechanisms in supervising, monitoring, and financial structure.

Administrative management describes tools used to ensure that patients are informed about the waiting time guarantee. They specify the obligation to inform patients in care contracts as well as when a care unit starts and at six months of operation. In addition, care providers are reminded of the obligation to inform patients as needed. As the care providers describe very sparse information provided to patients, it is reasonable to ask whether the tools used by administrative management are sufficiently effective. Waiting time guarantees are effective as a policy tool only when care providers are held accountable for achieving the targets and allowing patients to choose alternative care providers [17]. Administrative management does not accomplish the results they are expecting. The reliance on the diffusion of information, recommendations, and targets are known as soft governance [28]. The presence of contracts tries hard governance. However, this aspect of the contracts is not enforced with sanctions or rewards. In behavioral terms, interventions of administrative management are focused on antecedents rather than consequences [29]. The mechanisms that administrative management uses function as the antecedents of how information and support are given to patients. The behavior is reflected in the great degree of inconsistency in the information provided to patients.

Furthermore, administrative management relies on information from care providers. They describe how care providers ensure that they inform patients, but administrative management has few mechanisms to control whether this is the case. This increases the risk of care providers manipulating the information reported. Such behavior might not always be wrong, as the care provider may be doing so to save time and focus on other issues that are more pressing to patients. Further, the example of a care professional that printed referrals to avoid tracking by the system (seen in *Financial disincentives for efficient referral of patients*) imply that motivation to attaint to the care guarantee differ across care professionals. It is not solely determined by external incentives. However, the lack of correct reporting does undermine the funding administrative management’s ability to govern the care process towards increased equality in health. A different governance strategy could enable fuller control over the information given to patients by care providers.

In addition, economic mechanisms further undermine care providers’ willingness to inform patients. If care providers arrange care outside their own operation, they are, in some instances, mandated to carry the bill of care. This is typically more expensive than if the region had produced the care themselves. An earlier study predicted that this effect would decrease information given about the waiting time guarantee [8]. This study lends empirical support to the notion that economic incentives may hinder patients from being informed about the waiting time guarantee and provides an additional economic mechanism for waiting times. This mechanism has to do with the benefits of a queue. It is known that queues are beneficial to the care provider, as they ensure there will always be patients waiting to be treated. If the line is too short, the risk increases that there will soon be no one waiting. Consequently, the provider will have gaps in production and decreased incomes [11]. Further, long queues may motivate administrative management to alleviate production limits, which would enable larger profits for private care providers [11]. The current study illustrates how a provider refers patients within their own corporate group, contrary to the requirement of the contracts and law. This is probably done to ensure established queues. As there is an economic incentive to do this, the behavior can be viewed as created by the current incentive structure.

The current study carries lessons for any country with a present or future policy that requires providers to inform patients about their rights concerning waiting time guarantees. The current lack of patient information about the waiting time guarantee could be solved either by a redesign of the incentive structure, by relying on general practitioners to inform patients, or by providing patients with information without relying on care providers. Public information databases that require the patient to research the shortest waiting times and then negotiate a referral to the provider with the shortest waiting time require high degrees of health literacy. Any such solution risks increasing inequality in health. However, it is possible to require that the patient chooses a care provider using an IT platform that displays the waiting time [29, 30]. While such a system would make waiting times more equally distributed in the population, it carries a risk. If the system does not display all relevant health care quality indicators, it might not help patients at achieving good health, but only short waiting times. Patients with low health literacy still require the medical professional’s guidance in interpreting displayed health care quality indicators. In such situations the general practitioner may be most suited to inform the patients at time of referral. Interventions to improve information from general practitioners may be a fruitful route for ensuring equitable information access.

### Methodological considerations

This study highlights limitations in the information and support to patients and captures mechanisms that prevent communication processes about the waiting time guarantee. Several methodological limitations need consideration in interpreting the results.

This study was performed in the context of specialist care in Stockholm, Sweden. The challenges of waiting time guarantees are similar to those of other Nordic countries [8] and to OECD countries [17], but the specific reimbursement system and provider/purchaser model may limit transferability. This is countered by a rich context description in the methods section and a theoretical anchoring of the mechanisms described. Economic incentives and weak, soft governance are likely to prevent informed and supported patients in any context.

The data does not cover patients’ experiences of being informed, nor observations of the same, or systematically sourced documents (information to patients or guidelines for care providers or administrative management). However, previous studies align with the result that patients are generally not informed about the waiting time guarantee, making the present study’s data sources sufficient. Had the providers and administrative management reported a great degree of information and support to patients, documents and patient interviews would have been important data sources to make sure the information and support were not only given but also received. The most important contribution of this study is the hypotheses for mechanisms influencing the ability of care providers to inform. For that purpose, administrative management is the most important data source. However, for that aim, document analysis is still likely to have provided additional information.

The second and last authors collected the data, while the first author performed the analysis. Analyzing interview data without having collected it can create the risk that implicit information from the interviews was lost in analysis. This was countered by close collaboration with the authors, who performed the interviews to discuss ambiguities and clarify misunderstandings.

## Conclusions

This study investigated the information and support given to patients when the waiting time guarantee could not be fulfilled. The study confirmed a low degree of attainment in informing and supporting patients. Current governance and economic mechanisms seem insufficient in mobilizing healthcare to help patients benefit from the waiting time guarantee. If care providers are to carry the responsibility of informing patients about their rights, governance needs to be effective, and economic incentives need to be aligned.

## Appendix 1

### Consolidated criteria for reporting qualitative studies (COREQ): 32-item checklist

Tong A, Sainsbury P, Craig J. Consolidated criteria for reporting qualitative research (COREQ): a 32-item checklist for interviews and focus groups. *International Journal for Quality in Health Care*. 2007. Volume 19, Number 6: pp. 349–357.

**Table:** 

No. Item	Guide questions/description	Reported on Page # Description	
**Domain 1: Research team and reflexivity**			
*Personal Characteristics*			
1. Interviewer/facilitator	Which author/s conducted the interview or focus group?	HA, DE	
2. Credentials	What were the researcher’s credentials? E.g. PhD, MD	BCM: PhD Business Studies HA: PhD Medical Management DE: MD, MPH, PhD Physician HH:PhD	
3. Occupation	What was their occupation at the time of the study?	BCM: Research Assistant HA: Associate Researcher DE: Physician, Associate Researcher HH: Adjunct Professor	
4. Gender	Was the researcher male or female?	BCM: female HA: female HH: female DE: male	
5. Experience and training	What experience or training did the researcher have?	Researchers have extensive experience and training in qualitative research and interviewing. The interviewers had previous experience interviewing healthcare actors.	
*Relationship with participants*			
6. Relationship established	Was a relationship established prior to commencement of study?	The researchers had not met the participants before their interviews.	
7. Participant knowledge of the interviewer	What did the participants know about the researcher? e.g. personal goals, reasons for doing the research	The reason for the study was described in the e-mail used for recruiting participants and orally before the interview. Participants were aware of the interviewers’ roles in the project	
8. Interviewer characteristics	What characteristics were reported about the inter viewer/facilitator? e.g. Bias, assumptions, reasons and interests in the research topic	The information contained no personal details about the researchers or their interests. However, informants knew about the identity of the interviewers in advance and, thus, had the opportunity to read about interviewers’ research interests. The interviewers had no prior research experience within this research field. The consequence of the interviews may have been that the informants were asked to explain and clarify their answers further. Consequences for the quality of data collection should be mainly positive.	
**Domain 2: Study Design**			
*Theoretical framework*			
9. Methodological orientation and Theory	What methodological orientation was stated to underpin the study? e.g. grounded theory, discourse analysis, ethnography, phenomenology, content analysis	Page 3	
*Participant selection*			
10. Sampling	How were participants selected? e.g. purposive, convenience, consecutive, snowball	Page 4	
11. Method of approach	How were participants approached? e.g. face-to-face, telephone, mail, email	Page 4	
12. Sample size	How many participants were in the study?	Page 4	
13. Non-participation	How many people refused to participate or dropped out? Reasons?	Page 4	
*Setting*			
14. Setting of data collection	Where was the data collected? e.g. home, clinic, workplace	Page 5	
15. Presence of non-participants	Was anyone else present besides the participants and researchers?	No	
16. Description of sample	What are the important characteristics of the sample? e.g. demographic data, date	Page 4	
*Data collection*			
17. Interview guide	Were questions, prompts, guides provided by the authors? Was it pilot tested?	Page 5	
18. Repeat interviews	Were repeat interviews carried out? If yes, how many?	No	
19. Audio/visual recording	Did the research use audio or visual recording to collect the data?	Audio recording	
20. Field notes	Were field notes made during and/or after the interview or focus groups?	Yes	
21. Duration	What was the duration of the interviews or focus group?	Page 5	
22. Data saturation	Was data saturation discussed?	Yes	
23. Transcripts returned	Were transcripts returned to participants for comment and/or correction?	No	
**Domain 3: analysis and findings**			
*Data analysis*			
24. Number of data coders	How many data coders coded the data?	2	
25. Description of the coding tree	Did authors provide a description of the coding tree?	Appendix 2 provides a description of the coding steps	
26. Derivation of themes	Were themes identified in advance or derived from the data?	Page 5–6	
27. Software	What software, if applicable, was used to manage the data?	Page 5	
28. Participant checking	Did participants provide feedback on the findings?	No	
*Reporting*			
29. Quotations presented	Were participant quotations presented to illustrate the themes/findings? Was each quotation identified? e.g. participant number	Yes.	
30. Data and findings consistent	Was there consistency between the data presented and the findings?	Yes	
31. Clarity of major themes	Were major themes clearly presented in the findings?	Yes. From page 6 to 15	
32. Clarity of minor themes	Is there a description of diverse cases or discussion of minor themes?	Yes. From page 6 to 15	

### Standards for reporting qualitative research (SRQR) checklist

**Table:** 

No.	Topic	Item	Manuscript page no.
**Titel and Abstract**			
S1	Title	Concise description of the nature and topic of the study identifying the study as qualitative or indicating the approach (e.g., ethnography, grounded theory) or data collection methods (e.g., interview, focus group) is recommended	The title has been submitted in a separate file
S2	Abstract	Summary of key elements of the study using the abstract format of the intended publication, typically includes objective, methods, results, and conclusions	1
**Introduction**			
S3	Problem formulation	Description and significance of the problem/phenomenon studied, review of relevant theory and empirical work; problem statement	2–3
S4	Purpose or research question	Purpose of the study and specific objectives or questions	3
**Methods**			
S5	Qualitative approach and research paradigm	Qualitative approach (e.g., ethnography, grounded theory, case study, phenomenology, narrative research) and guiding theory if appropriate; identifying the research paradigm (e.g., positivist, constructivist/interpretivist) is also recommended	3
S6	Researcher characteristics and reflexivity	Researchers’ characteristics that may influence the research, including personal attributes, qualifications/experience, relationship with participants, assumptions, or presuppositions; potential or actual interaction between researchers’ characteristics and the research questions, approach, methods, results, or transferability	COREQ
S7	Context	Setting/site and salient contextual factors; rationale*	3–4
S8	Sampling Strategy	How and why research participants, documents, or events were selected; criteria for deciding when no further sampling was necessary (e.g., sampling saturation); rationale*	4–5
S9	Ethical issues pertaining to human subjects	Documentation of approval by an appropriate ethics review board and participant consent, or explanation for lack thereof; other confidentiality and data security issues	17
S10	Data collection methods	Types of data collected; details of data collection procedures including (as appropriate) start and stop dates of data collection and analysis, iterative process, triangulation of sources/methods, and modification of procedures in response to evolving study findings; rationale*	5
S11	Data collection instruments and technologies	Description of instruments (e.g., interview guides, questionnaires) and devices (e.g., audio recorders) used for data collection; if/how the instrument(s) changed over the course of the study	5
S12	Units of study	Number and relevant characteristics of participants, documents, or events included in the study; level of participation (could be reported in results)	4
S13	Data processing	Methods for processing data prior to and during analysis, including transcription, data entry, data management and security, verification of data integrity, data coding, and anonymization / deidentification of excerpts	5
S14	Data analysis	Process by which inferences, themes, etc., were identified and developed, including researchers involved in data analysis; usually references a specific paradigm or approach; rationale*	5–6
S15	Techniques to enhance trustworthiness	Techniques to enhance trustworthiness and credibility of data analysis (e.g., member checking, audit trail, triangulation); rationale*	5, 16
**Results/Findings**			
S16	Synthesis and interpretation	Main findings (e.g., interpretations, inferences, and themes); might include development of a theory or model, or integration with prior research or theory	6–13
S17	Links to empirical data	Evidence (e.g., quotes, field notes, text excerpts, photographs) to substantiate analytic findings	6–13
**Discussion**			
S18	Integration with prior work, implications, transferability, and contribution(s) to the field	Short summary of main findings; explanation of how findings and conclusions connect to, support, elaborate on, or challenge conclusions of earlier scholarship; discussion of scope of application/generalizability; identification of unique contribution(s) to scholarship in a discipline or field	13–15
S19	Limitations	Trustworthiness and limitations of findings	16
S20	Conflicts of interest	Potential sources of influence or perceived influence on study conduct and conclusions; how these were managed	17
S21	Funding	Sources of funding and other support; role of funders in data collection, interpretation, and reporting	17

* The rationale should briefly discuss the justification for choosing that theory, approach, method, or technique rather than other options available, the assumptions and limitations implicit in those choices, and how those choices influence study conclusions and transferability. As appropriate, the rationale for several items might be discussed together.

## Appendix 2

### Thematic analysis - steps followed

**Table:** 

Step	Description	Example from analysis in the present study
1) Become familiar with the data	Read the data several times. Rough notes and early impressions	Notes: - Referral is sent to another care provider when the waiting time guarantee cannot be fulfilled - The patient receives written information about a referral sent to another care provider
2) Generation of initial codes	Open coding. Systematic and meaningful organization of data to answer research questions	Code: *Information in brochures, letters, and pamphlets* - “If we forward the referral, then that patient will receive a letter stating that we have received the referral and it has been assessed by a urologist, in this case, who has decided that this referral can be forwarded to another care provider”
3) Search for themes	Grouping codes into initial themes and subthemes	Forming tentative themes and subthemes (here with examples of codes): *Different ways to inform patients about the waiting time guarantee* • Oral info to patients when they call • Information in brochures, letters, and pamphlets • Unclear how patients receive information about the waiting time guarantee *Further referral when the waiting time guarantee cannot be fulfilled* • Referrals from the waiting time guarantee office • Patients may ask the referrer or receptionist to send a referral elsewhere • Patients can contact the Waiting time guarantee Office or another care provider
3) Reviewing	Check if themes and subthemes represent their codes and all relevant data	Merging subthemes and moving codes: • Subthemes *The patients is responsible for a new referral* and *The care provider is responsible for a new referral* were merged in one theme *Inconsistent support to patients* • Subthemes *Do not inform about the waiting time guarantee because they can fulfill the waiting time guarantee* and *No info for patients about the waiting time guarantee due to the short waiting time* were merged in one subtheme *No info for the patient* • Subtheme *Collaboration between care providers* was divided into two categories *Between different healthcare and units* and *Between the same unit*
4) Defining	Renaming themes and assessment of different issues	Renaming: • *Further referral when the waiting time guarantee cannot be fulfilled* was renamed as *Inconsistent support to patients.* • *Different ways to inform patients* was renamed as *Inconsistent information to patients* Assessment of issues such as: • If the data support themes and it makes sense • If one theme contains to many subthemes • If there are overlapping themes

## Data Availability

The datasets generated and/or analyzed during the current study are not publicly available due to the integrity of the participants but are available from the corresponding author on reasonable request.
